# Prevention of Motorcycle–Car Door Collisions by Using a Deep-Learning-Based Automatic Braking Assistance System

**DOI:** 10.3390/s26072175

**Published:** 2026-03-31

**Authors:** Yaojung Shiao, Tan-Linh Huynh

**Affiliations:** 1 Department of Vehicle Engineering, National Taipei University of Technology, Taipei 10608, Taiwan; yshiao@ntut.edu.tw; 2 Railway Vehicle Research Center, National Taipei University of Technology, Taipei 10608, Taiwan

**Keywords:** automatic braking assistance system, braking intensity, car door states, front braking ratio, initial braking speed, preventable accidents, YOLOv12s object detection model

## Abstract

Collisions between motorcycles and car doors that are being opened are common, preventable accidents that can result in fatalities. A critical limitation of safety advancements in both cars and motorcycles is high cost associated with the use of radar sensors. In this study, a deep learning model was integrated into an inexpensive and camera-utilizing automatic braking assistance system for motorcycles to enhance braking performance and alert motorcyclists to avoid collisions. This research involved two stages: (1) the training of a deep learning model for detecting car door states and (2) the design of safety mechanisms for selecting appropriate braking intensity and front braking ratio values on the basis of the model’s output, time-to-collision, the rider’s braking action, and the initial braking speed, in order to achieve optimal braking performance. Specifically, the YOLOv12s object detection model showed high performance in predicting the states of car doors, exhibiting precision, recall, and mean average precision values of 90.5%, 80.6%, and 87.8%, respectively. The braking intensity of the system was set to 0%, 25%, 50%, or 100% in scenarios involving opening states of the car door (closed, small, medium, or large opening), time-to-collision values, and the rider’s braking action. The optimal front braking ratio function was determined based on the initial braking speed to achieve the optimal braking performance. At an initial braking speed of 60 km/h, the braking stroke under a front braking ratio of 45% was 35.61% and 13.37% shorter than those under front braking ratios of 20% and 60%, respectively. The proposed braking assistance system can feasibly be deployed in the real world because it can respond within a safe time window under the conditions studied, which is approximately 0.5 s. However, further refinement is required, including improvement of the robustness of the object detection model through the collection of a larger and more diverse dataset, experimental measurement of front braking ratios to determine the optimal braking performance in real scenarios, and design of a physical actuator to control braking intensity and the front braking ratio in real time.

## 1. Introduction

Motorcyclists are vulnerable road users, and their safety is influenced by motorcycle-related factors, such as braking performance and maximum speed; rider-related factors, such as balance, reaction skills, and protective equipment use; environmental conditions, such as rain, wind, and fog; and infrastructure-related factors, such as one- versus two-way traffic, road surface quality, the existence of dedicated motorcycle lanes, and road geometry [1]. In Europe, more than 3500 motorcyclists died in traffic accidents in 2019, and motorcyclists had a 9–30 times higher risk of a fatal traffic injury compared with car drivers [2]. Moreover, the overall risk is increasing because of global increases in motorcycle ownership [3] and the number of motorcyclists [4]. In Taiwan, a large number of motorcycles operate on roads [5], and motorcycle accidents are common. In 2023, Taiwan recorded 423,786 motorcycle-related accidents, which resulted in 1886 motorcyclist deaths, with these values representing 18.19% and 11.27% increases, respectively, compared with the corresponding values for 2019. Cars in Taiwan are often allowed to park on both sides of the road, which contributes to the risk of accidents involving collisions between an approaching motorcycle and a car door being opened; 1171 such accidents were recorded in Taiwan in 2023 [6].

Safety systems on vehicles have been developed to prevent accidents involving motorcycles and car door openings. For cars, conventional vehicles typically engage an automatic door-lock system when the vehicle is traveling above a certain speed (usually around 20 km/h). When cars are parked, car users observe directly or through the side-view mirror to check for approaching pedestrians or vehicles before opening the doors. However, this method cannot accurately estimate the time interval before an approaching vehicle arrives, and it relies solely on the users’ visual judgment and experience. Zhung et al. [7] proposed an anti-collision system that uses a single digital camera and a Field Programmable Gate Array chip mounted on the side-view mirror to detect approaching vehicles, estimate their speed, and give a warning when it exceeds a threshold (i.e., 16 km/h). Similarly, Huang [5] proposed a fuzzy-logic-based, car-door-opening control model that detects the approaching object, estimates its speed and distance to the car using short-wave radar sensors, and gives a warning through three hazard modes: danger, caution, and warning. For motorcycles, research on collision prevention is limited. Pfeiffer et al. [8] developed an emergency braking assistance system that uses radar sensors to detect potential collisions with vehicles, pedestrians, or obstacles ahead, alerting the rider and assisting with braking when necessary. Although radar-based brake-assistance systems support motorcyclists effectively under various weather and nighttime conditions, they cannot accurately capture the shape or opening state of a car door and therefore cannot always take timely precautionary measures.

Recently, artificial intelligence (AI) has been widely adopted in transportation applications due to its effectiveness [9]. Various object detection methods have been applied in traffic safety, including motion-based approaches (e.g., frame differencing, optical flow, and background modeling) and appearance-based approaches (e.g., handcrafted-feature models, CNN-based models, and transformer-based models) [10,11]. Building on these advances, this study develops a deep-learning model that predicts car-door openings ahead of a motorcycle using camera input to prevent motorcycle-car door collisions. Cameras offer detailed visual information that allows the system to capture object characteristics, such as shape, texture, and door-opening status.

Beyond advances in hazard-alert systems, safety systems also involve enhanced braking technologies in motorcycles that directly prevent or mitigate collisions. Separate brake systems are common in classic motorcycles. In such systems, braking performance depends heavily on the rider’s skill [12,13,14,15,16]. Specifically, optimal braking is achieved when both the front and rear brakes are applied simultaneously. However, approximately one-third of riders were found to use only one of the brakes, indicating poor riding skills [16]. Advanced braking systems have been developed, gradually replacing the conventional separate brake systems. Tani et al. [16] proposed a combined brake system for small motorcycles, which was discovered to achieve a 34% shorter stopping distance than that achieved using the rear brake alone. Mortimer et al. [15] demonstrated that motorcycles equipped with integrated brake systems achieved a 30–50% increase in mean deceleration on dry pavements compared with motorcycles having separate brake systems. Dinges et al. [17] compared the performance of motorcycles with and without an antilock braking system, finding that this system increased mean deceleration by 27%. In general, such systems help motorcyclists use both the front and rear brakes simultaneously during braking, thereby enhancing braking performance.

Notably, collisions between motorcycles and opening car doors are likely to occur when riders have insufficient time to brake efficiently, even with advanced braking systems. This underscores the importance of timely intervention either through automatic braking assistance or by alerting the rider early enough to take actions. Therefore, we proposed an automatic braking assistance system that applies both the front and rear brakes simultaneously for low-powered motorcycles used on city and urban roads, incorporating a deep-learning-based object detection and a safety mechanism of automatic braking intervention in consideration of the rider’s action and the hazard level.

## 2. Concept of the Proposed Automatic Braking Assistance System

**Figure 1:**
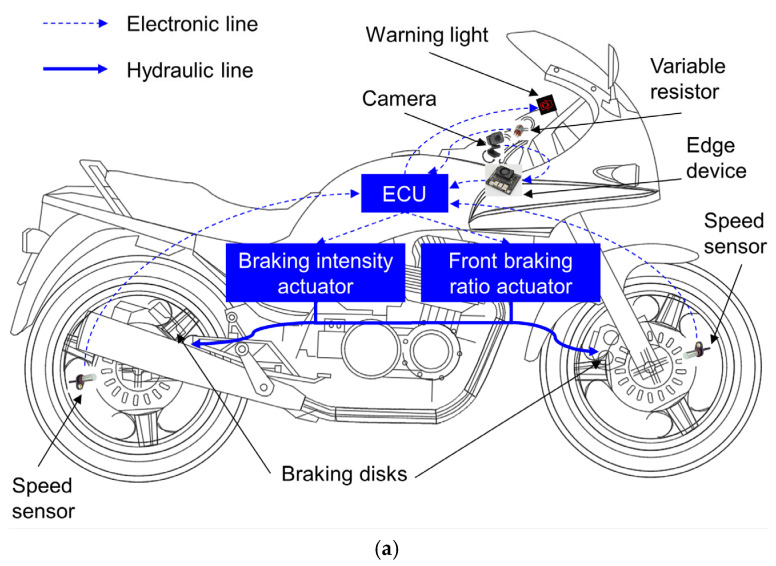

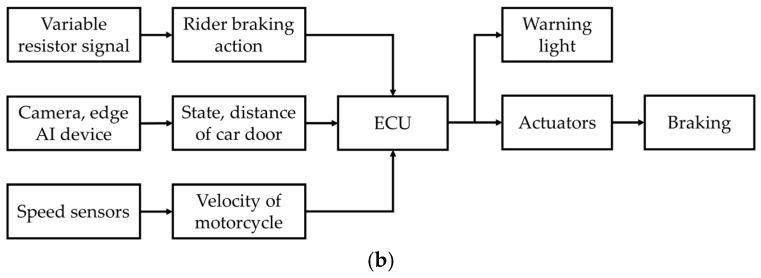
The proposed automatic braking assistance system (**a**) configuration and (**b**) diagram.

The proposed automatic braking assistance system is designed to be installed on a low-powered motorcycle, as shown in Figure 1a. This system is integrated into a conventional separate hydraulic brake system, with the addition of the following key components: a high-resolution camera, an edge AI device, two angular speed sensors, two variable resistors, an actuator with adjustable braking intensity, an actuator with adjustable braking ratio, three warning lights, and an electronic control unit (ECU). The camera is mounted at the center of the handlebars to capture signals from both sides of the motorcycle. In general, a higher camera speed is desirable. However, it is constrained by cost, as faster cameras are typically more expensive. Additionally, the camera speed should be aligned with the processing capability of the edge AI device to optimize the system’s overall processing time. For example, the Jetson Orin Nano 8 GB Developer Kit, which is widely used in AI-based applications, achieves an inference time of 30.58 ms/image (equivalent to 32.7 frames/s) when running the YOLOv12s model with an input resolution of 1280 × 1280 pixels [18]. Therefore, we selected a camera with a compatible frame rate of 30 frames/s (equivalent to 33.33 ms/image). The variable resistors are mounted to the joint of the handlebar and brake lever to determine the rider’s braking action. On the other hand, the field of view should be chosen to detect opening car doors that pose a direct and significant hazard to the motorcycle while excluding those that do not. If the field of view is too wide, the system may capture objects that are far to the sides and do not present any real danger. Conversely, if it is too narrow, the system may fail to detect nearby objects that pose a hazard. The required field of view is determined based on the extreme scenario in which the camera needs to detect the entire door at the closest possible distance, specifically, when the front wheel of the motorcycle approaches the left edge of the opening car door. Based on reference [19], the maximum door opening width for a typical sedan ranges from 762 to 1067 mm; for this study, we selected a representative width of 800 mm. The distance between the motorcycle’s handlebars and the opening car door ahead is estimated to be approximately 1000 mm. Therefore, to capture the full shape of the opening car door, the minimum field of view was estimated to be approximately 39 degrees on one side, or 78 degrees in total for both sides. In this study, we selected a Raspberry Pi AI camera with a field of view of 78.3 degrees and a speed of 30 frames/s [20], which meets all of the stated assumptions.

The angular speed sensors measure the rotational speed of the front and rear wheels. The ECU receives the prediction signal from the edge AI device, combines it with distance signals from camera, speed signals from the speed sensors and the rider action from the variable resistor, and then controls the brake actuators while also warning the motorcyclist of potential danger. The warning lights on the dashboard indicates the degree of hazard posed by car doors opening ahead. The actuator with adjustable braking intensity modifies the braking force, and the actuator with adjustable braking ratio modifies the braking distribution between the front and rear brakes.

The operating principle of the automatic braking assistance system is illustrated in Figure 1b. The camera captures road images and sends the frames to the edge AI device, where a deep-learning model detects the opening state of the car door ahead (closed, small opening, medium opening, or large opening). Based on the speed sensors on the motorcycle’s front and rear wheels, the motorcycle’s mean velocity is obtained. The distance between the motorcycle and the opening car door is estimated using monocular vision methods with a camera. It can be achieved through image-processing algorithms, deep-learning-based depth estimation, or instance-segmentation approaches with camera focal-length information [21,22,23,24]. Using the estimated distance and the motorcycle’s velocity, the time-to-collision (TTC) is calculated according to Equation (1). The rider’s braking action (yes or no) is defined through signals change from the variable resistor mounted on the brake levers. Once the ECU determines the car-door opening state, the TTC, and the rider’s action, it evaluates the hazard level and provides an appropriate response. First, it activates warning lights corresponding to the opening levels of the car door to alert the motorcyclist. Then, when necessary, the ECU engages the braking through actuators to achieve effective braking performance. When the TTC is greater than a predefined threshold, the automatic braking assistance system does not apply braking; it only provides visual warnings to the rider. If the rider does not take actions in response to the hazard, the system progressively applies braking. However, if the rider initiates braking, the braking response is fully under the rider’s control, and the automatic braking intervention is no longer applied.

(1) $$TTC(t)=\frac{X(t)}{V(t)}$$

where *TTC*(*t*) is time-to-collision at instant *t*, *X*(*t*) is the estimated distance at instant *t* from the camera mounted on the motorcycle to the opening door of the parked car, and *V*(*t*) is the velocity of the motorcycle at instant *t*.

**Figure 2:**
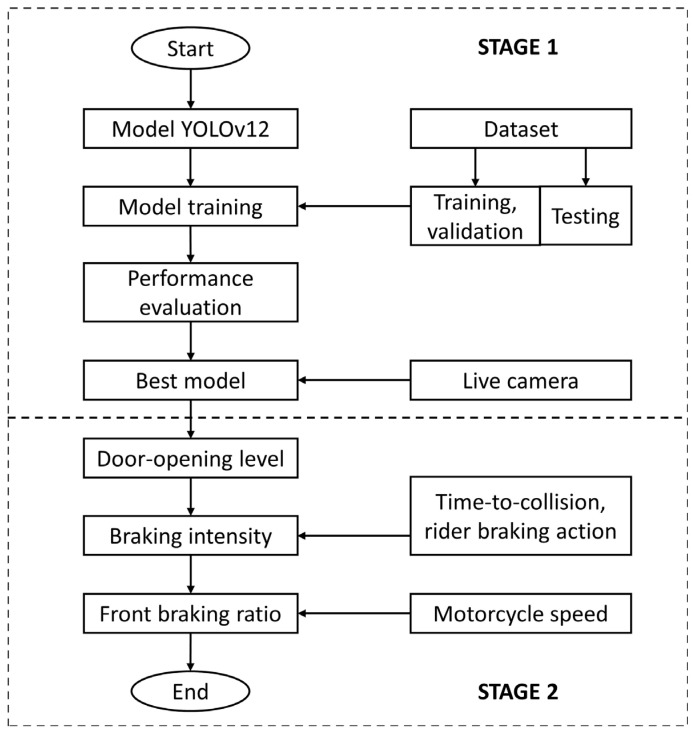
The implementation stages of the proposed method.

The manuscript was constructed in two stages in Figure 2. The first stage involved the implementation of a deep learning model for detecting the state of car doors ahead of a motorcycle. Subsequently, the second stage involved the establishment of a method for automatically determining the appropriate braking intensity and front braking ratio.

## 3. Development of Deep-Learning Models to Detect Car Door Opening States

This section focuses on the first stage of the study, which involved identifying the most suitable deep learning model for detecting the opening state of car doors. It describes the adopted dataset collection and labeling processes, the architecture of the selected deep-learning-based object detection model, and the validation of the selected model.

### 3.1. Data Collection and Labeling for Car Door Opening States

A conventional hinged car door is generally understood to be in one of two basic physical stages: closed or open [25]. The closed state indicates that the door is fully shut and latched to the car frame. In this position, the door is aligned with the frame, and therefore, the door-opening angle is 0°. The open state indicates that the door is no longer latched to the car frame. The maximum door-opening angle corresponds to the fully open position, i.e., the maximum extent to which a door can swing outward. For most typical sedan vehicles, this maximum angle is commonly around 75° to 90° [26]. In this study, we consider a large opening angle for both front and rear doors to be >40°. In real traffic situations, a widely opened car door can pose a significant hazard to approaching vehicles, whereas a slightly opened door may not necessarily cause danger because the rider still has sufficient space to maintain a safe distance or avoid the obstacle. Therefore, in this study, the door-opening state was categorized into three levels: small, medium, and large, to support appropriate actions and smooth responses from the automatic braking system. Accordingly, the car door was classified into one of four states: closed (0°), small opening (<10°), medium opening (10–40°), and large opening (>40°).

Given that no such data previously existed, the present study collected data on car door states by using high-resolution cameras through two approaches: (1) filtering images showing car door states from road-recording videos captured by vehicle-mounted cameras and (2) photographing the car door states of cars parked on roads or at exhibition centers. The collected data covered various road types in Taiwan (e.g., urban, suburban, and rural roads); multiple commercial car segments from various brands, such as Toyota, Mercedes-Benz, and BMW; and different times of day over several months. All the collected data were double-checked by an experienced PhD researcher and reviewed in consultation with the professor regarding any discrepancies. To ensure the diversity and robustness of the dataset, augmentation methods, including rotation, scaling, translation, hue, saturation, and value (HSV) adjustment, mixup, cutmix, and mosaic, were applied [27]. Specifically, scaling improves the model’s capability in detecting the opening doors from far or close distances and at different sizes. HSV adjustment improves the detection capability at different light and weather conditions. Translation, mixup, cutmix, and mosaic improve the detection capability for the opening doors that are partially obscured. In fact, in real-world situations, motorcyclists typically ride slowly during such adverse weather and can thus manage door-opening risks more effectively.

**Figure 3:**
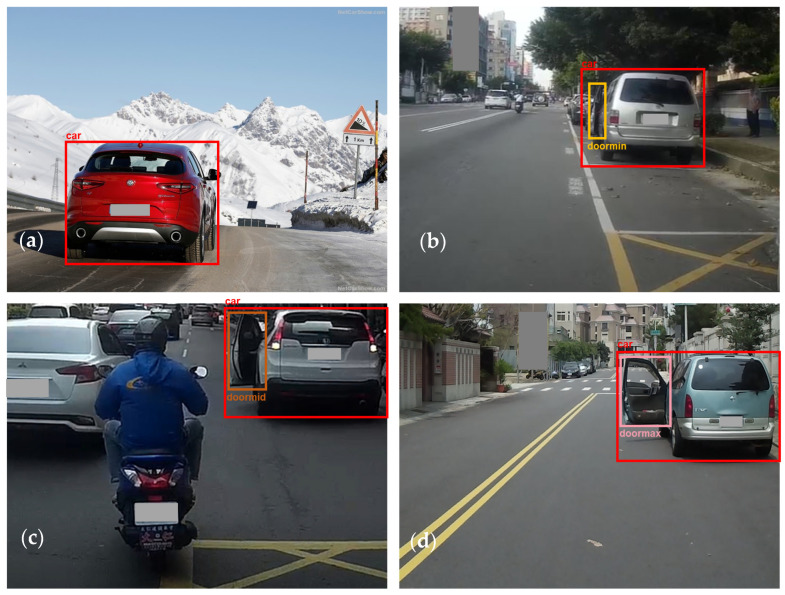
Examples for car door states in the dataset: (**a**) closed, (**b**) small opening, (**c**) medium opening, and (**d**) large opening.

After the images were collected, they were classified on the basis of the defined car door states and labeled as follows: small-angle openings were labeled “doormin”, medium-angle openings were labeled “doormid,” and large-angle openings were labeled “doormax”. In addition, all cars were also labeled “car”. Example images in the dataset for the four types of labels are presented in Figure 3.

The collected dataset, which comprised 1485 images containing 2398 labels across four classes (i.e., “car”, “doormin”, “doormid”, and “doormax”), was used to train deep learning models to detect car door states. The dataset was divided into training and validation sets at an 80:20 ratio, and these sets comprised 1190 and 295 images, respectively (Table 1). Additionally, we tested the performance of the trained deep learning model on 15 unseen images.

### 3.2. Deep Learning Model for Detecting Car Door States

#### 3.2.1. Architecture of YOLOv12

Object detection is a critical task in modern computer vision and has diverse applications in areas such as education, agriculture, industry, health care, and transportation. The YOLO (You Only Look Once) series of models consistently outperforms other computer vision approaches in terms of object detection capabilities [28]. The original YOLO framework, introduced by Redmon et al. [29], can process images in real time at 45 frames per second with high prediction accuracy. The original YOLO framework has been continually improved to increase its accuracy and inference speed to meet the requirements of real-time applications. The YOLOv12 framework has been empirically demonstrated to have higher prediction speeds and mean average precision than earlier YOLO versions do owing to the advanced modules in its backbone, neck, and head [30]. Specifically, the backbone of YOLOv12 employs the Residual Efficient Layer Aggregation Network (R-ELAN) module, which is combined with 7 × 7 separable convolutions and multiscale feature pyramids to enable the detection of small or complex objects. The neck of YOLOv12 incorporates an area attention mechanism powered by FlashAttention to focus on critical regions within images, improve detection in cluttered or dynamic environments, and reduce memory transfers and computational overhead. Furthermore, the head of YOLOv12 optimizes loss functions, bounding box regression, streamlined multiscale detection pathways, and data augmentation. Because of these incorporations, YOLOv12 achieves higher precision in object localization, faster convergence, and more robust object detection than other YOLO versions do [31].

**Figure 4:**
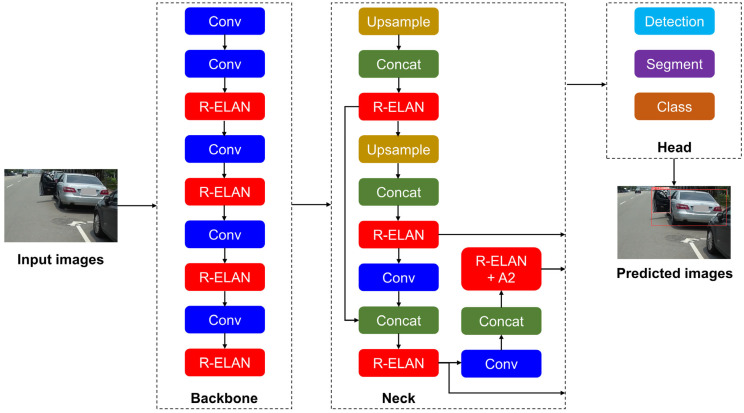
YOLOv12 architecture.

In this study, a deep-learning-based object detection model was used to detect car door states, representing the first stage in the development of an automatic braking assistance system for motorcycles. Once car door states have been detected, the proposed automatic braking assistance system rapidly determines whether the motorcycle should brake; thus, real-time processing is essential. YOLOv12 was thus selected as the object detection model. Figure 4 displays the architecture of YOLOv12.

#### 3.2.2. Metrics for Evaluating the Performance of YOLOv12

A diverse set of evaluation metrics can be used to assess the effectiveness of YOLOv12 in within-image object detection, classification and localization. The primary metrics employed are precision, recall, and mean average precision (mAP). In the context of this study, Precision represents the proportion of correctly identified objects among all positive predicted objects made by the YOLOv12 model [Equation (2)]. Recall, also referred to as the true positive rate, measures the proportion of correct predictions relative to the total number of actual objects [Equation (3)]. Average precision (AP) is the area under the precision–recall curve, representing the accuracy of predictions for a single category. AP is expressed in Equation (4), where *K* represents a set of thresholds and $R_{i}$ and $P_{i}$ denote the recall and precision at each threshold *i*. mAP is the average of AP values across all categories and ranges from 0 to 1 [Equation (5)]. TP, FP, and FN refer to the numbers of true positives (correct detections of existing objects), false positives (incorrect object detections), and false negatives (missed detections of existing objects), respectively.

(2) $$Precision=\frac{TP}{TP+FP}$$

(3) $$Recall=\frac{TP}{TP+FN}$$

(4) $$AP=\sum_{i=1}^{K}\left(R_{i}-R_{i-1}\right)P_{i}$$

(5) $$mAP=\frac{\sum AP}{Num(class)}$$

### 3.3. Verification of the YOLOv12 Model

#### 3.3.1. Setup

The collected dataset was used to train deep learning models of various scales from the YOLOv12 family (YOLOv12n, YOLOv12s, YOLOv12m, and YOLOv12l). These models were executed on a personal computer equipped with an AMD Ryzen 9 5950X 16-core processor (3.40 GHz) and an NVIDIA GeForce RTX 3080 graphics processing unit (10 GB). Several crucial augmentation methods—including scaling, translation, flipping, HSV adjustment, mosaic, etc.—were applied during the model training process. Moreover, parameters such as image size, batch size, and number of epochs were specified in the model training. The hyperparameters of the YOLOv12 models, along with the computer configuration, are detailed in Table 2.

#### 3.3.2. Performance Evaluation

**Figure 5:**
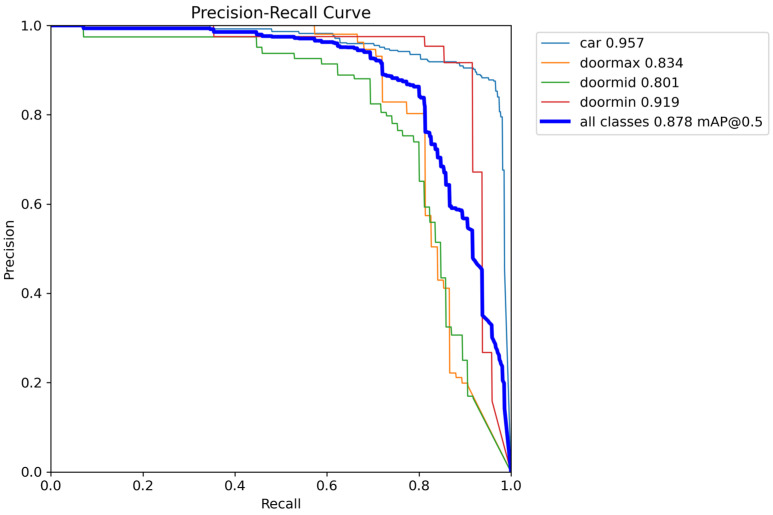
Precision–recall curves for four classes using the YOLOv12s model.

Table 3 presents the performance of YOLOv12n, YOLOv12s, YOLOv12m, and YOLOv12L in the detection of car door states. The precision, recall, and mAP@0.5 (mAP at an Intersection-over-Union threshold of 0.5) values across four classes (i.e., car, doormin, doormid, and doormax) are listed in the table. All models exhibited high performance, indicating their effectiveness in detecting car door states. Among the four models, YOLOv12s exhibited the best performance, achieving the highest mAP@0.5 value as well as high precision and recall. Moreover, YOLOv12s has considerably fewer parameters than do YOLOv12m and YOLOv12L, and its average inference time per frame was substantially shorter than those of YOLOv12m and YOLOv12L. Thus, among the examined models, YOLOv12s is easier and more suitable to deploy in real-time object detection applications. Consequently, this model was adopted in the automatic braking assistance system developed in the present study. The average mAP@0.5 values obtained for all classes with the YOLOv12s model are illustrated in Figure 5 in the form of precision–recall curves. Additionally, a comparison of the selected YOLOv12s model with and without augmentation methods is presented in Table 4, demonstrating that augmentation methods substantially enhance the model’s performance.

#### 3.3.3. Prediction Results of the YOLOv12s Model

The effectiveness and robustness of the trained YOLOv12s object detection model were further validated by testing its prediction performance on 15 unseen images. This test set covered all car door stages, with each stage represented by a relatively similar number of images (i.e., 3 to 4 images). These images were labeled and input into the trained YOLOv12s model for car door state prediction. The model’s performance metrics were as follows: precision = 0.961, recall = 0.903, and mAP@0.5 = 0.938, indicating high prediction capability on the test set. However, misclassification still occurred in some cases; for example, the model failed to detect a large opening stage when the door was partially obscured by a person stepping out of the car. A larger and more diverse dataset is needed to further improve the model’s generalizability in real-world conditions.

**Figure 6:**
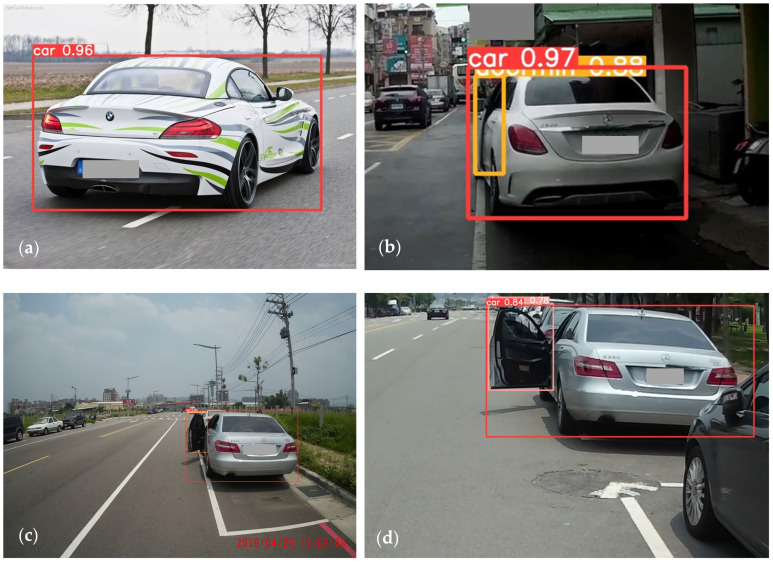
An example of the prediction results for car door states from the trained YOLOv12s model: (**a**) closed, (**b**) small opening, (**c**) medium opening, and (**d**) large opening.

An example of the prediction results is presented in Figure 6, which shows the object detection model’s correct high-confidence predictions of four states for car doors on roads. The high-performance YOLOv12s model is suitable for the first stage of the proposed automatic braking assistance system because it can accurately detect and warn motorcyclists about scenarios involving car door states ahead. The next section details the optimal braking strategy for the proposed system.

## 4. Selection of Braking Intensity

The second stage of the proposed system involves the selection of braking intensity, which is implemented by adjusting the oil pressure in the separate front and rear brake systems. A policy for a formalized safety mechanism for the automatic braking assistance system is present in Table 5. The system consistently warns the rider through lights corresponding to the door-opening level (green for small, yellow for medium, and red for large). At any time after the door opening is detected, if the rider responds to the collision risk by applying the brakes manually, the braking response remains fully under the rider’s control, and automatic braking is no longer applied. The automatic braking assistance system is activated when there is no rider intervention in the following cases: (1) When TTC decreases from 3 s to 1.5 s, the system applies 25% of the maximum braking intensity for medium or large door-opening levels to slightly reduce the motorcycle’s velocity and strengthen the warning to the rider; and (2) When TTC decreases below 1.5 s, the system takes braking control for medium and large openings by applying 50% or 100% of the maximum braking intensity, respectively, to reduce the severity of a potential collision.

## 5. Analysis for Front Braking Ratio

This section details how the best braking performance can be achieved by selecting an appropriate front braking ratio in accordance with the motorcycle’s speed. The front braking ratio was defined as the braking force of the front brake relative to the total braking force. A motorcycle rider model and braking conditions were modeled in simulation software, and on the basis of the simulation results, an interpolation process was used to derive a function of the optimal front braking ratio. This section incorporates several figures and parameters from an earlier study in our lab, which has been made publicly accessible through reference [32].

### 5.1. Setup of the Simulation Model

**Figure 7:**
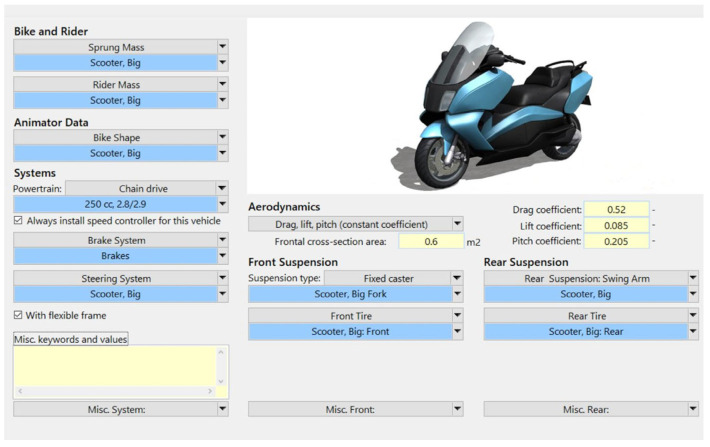
Motorcycle model setup in BikeSim software [32].

**Figure 8:**
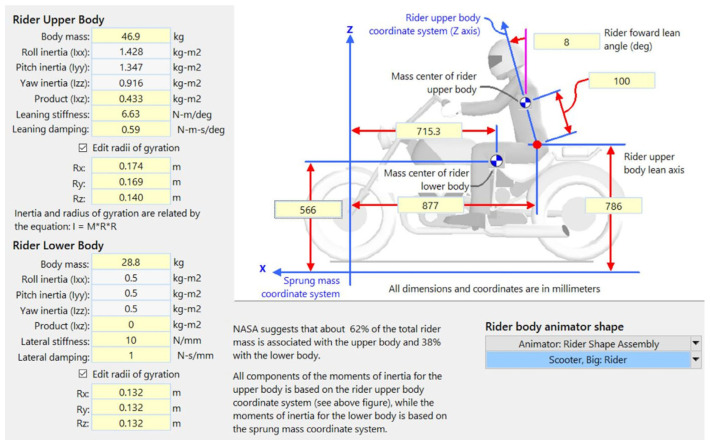
Rider model setup in BikeSim software [32].

Scooters are a widely used type of motorcycle in Taiwan. This study considered a scooter equipped with a hydraulic disk braking system and separate front and rear brakes. The key parameters and systems of the scooter are presented in Table 6. The scooter has an engine with a cylinder capacity of 250 cubic centimeters (cc), generating a rated power of 22 horsepower (hp). The scooter also has a telescopic fork for the front suspension and a unit swing arm for the rear suspension. BikeSim software (version 2019.1) offers the most precise, comprehensive, and efficient solutions for simulating the performance of two-wheeled vehicles. Supported by more than 20 years of real-world validation, this software is widely recognized as the leading tool for analyzing motorcycle dynamics, developing active control systems, and evaluating overall motorcycle performance [33]. Therefore, the scooter parameters and systems were modeled in BikeSim (Figure 7). Figure 8 depicts the motorcyclist model created in BikeSim, which was defined by mass, inertia, center of mass, stiffness, and damping of the upper and lower rider components.

**Figure 9:**
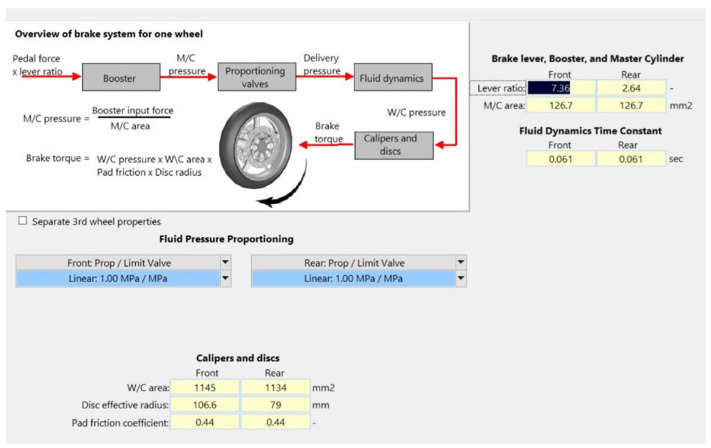
Braking torque transmission flowchart [32].

Boundary conditions for braking—including braking torque, road profile, initial speed, and front braking ratio—are embedded into BikeSim. The braking torque transmission flowchart is shown in Figure 9. The maximum braking torque that can be applied to each front and rear wheel is 624 Nm, corresponding to a motorcyclist’s handle force of 200 N. For the reasons mentioned in Section 3.1, a dry asphalt road profile was used in this study. The initial braking speed indicates the motorcycle’s speed when braking begins. This study used specific initial braking speeds that were defined on the basis of three common motorcycle operating speeds, namely 30, 45, and 60 km/h. These speeds correspond to narrow urban, standard urban, and suburban roads, respectively. For each speed, front braking ratios ranging from 20% to 60% were tested in increments of 2.5%. Finally, braking stroke, defined as the distance from the onset of motorcycle braking to a complete stop, was used as a braking performance metric [32].

### 5.2. Interpolation of Braking Simulation Results

**Figure 10:**
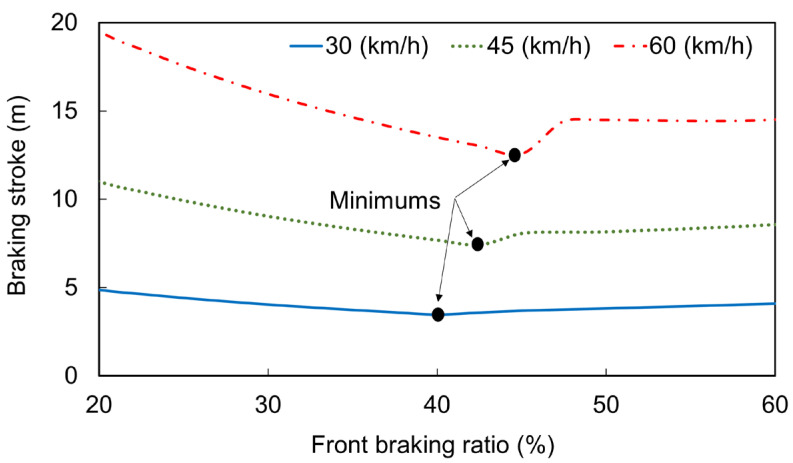
Simulation results for the braking stroke under different front braking ratios at three initial braking speeds [32].

Figure 10 presents the braking simulation results for the three initial braking speeds under various front braking ratios. For each initial speed, the braking performance varied with the front braking ratio. Performance was poor when the ratio was small or large. For instance, at an initial speed of 60 km/h, the braking stroke under a front braking ratio of 45% was 35.61% and 13.37% shorter than those under front braking ratios of 20% and 60%, respectively. For initial speeds of 30, 45, and 60 km/h, the best braking performance was achieved when the front braking ratio was 40%, 42.5%, and 45%, respectively [32].

**Figure 11:**
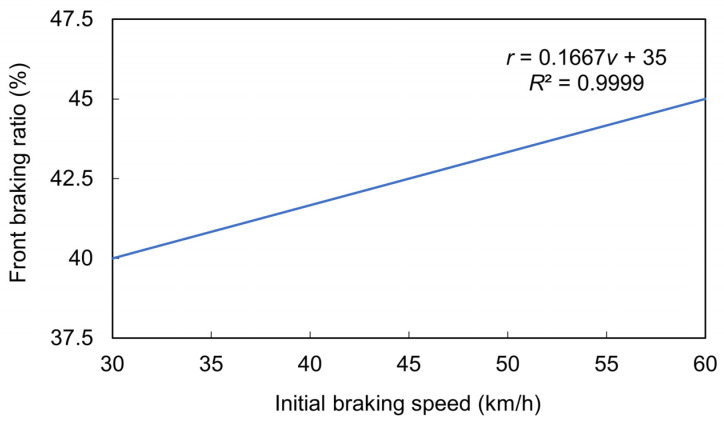
The interpolated association between the optimal front braking ratio and the initial braking speed for optimal braking performance.

**Figure 12:**
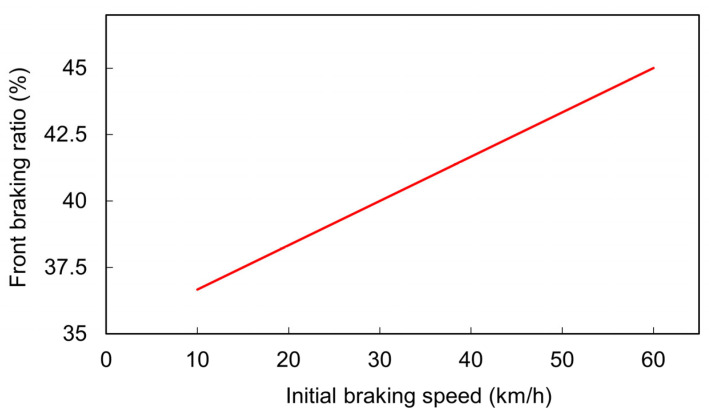
The extrapolated association between the optimal front braking ratio and the initial braking speed for optimal braking performance.

On the basis of the aforementioned results, the interpolated association between the optimal front braking ratio and the initial braking speed was derived (Figure 11). To achieve the optimal braking performance, a higher front braking ratio is required under a higher initial braking speed. Interpolation was conducted on the basis of the three best performance points to derive a function describing the relationship between the front braking ratio and the initial speed over the speed range of 30–60 km/h; the function is expressed in Equation (6). Given real-world motorcycle operating conditions, Equation (6) can be extrapolated to initial braking speeds ranging from 10 to 60 km/h. Over this speed range, the optimal front braking ratio for the automatic braking assistance system changes from 36.67% to 45.0% (Figure 12).

*r* = 0.1667*v* + 35, (6)

where *v* is the initial braking speed, and *r* is the optimal front braking ratio.

## 6. Limitations and Future Research

The present study is one of the first studies on developing a safety system for motorcycles with the application of deep learning. Although the study demonstrates that the proposed automatic braking assistance system is highly feasible, the study had some limitations. First, the study lacks experimental validation. However, full physical experimentation would pose safety hazards at this early stage of development. Meanwhile, BikeSim is a widely validated and commonly used simulation platform in vehicle dynamics research. Second, the function representing the association between the front braking ratio and the initial braking speed was derived from a limited number of data points, which may restrict the generalizability of our results. To enhance the robustness of the method, more data points are necessary to cover a wider range of velocities. Third, the braking-intensity thresholds used in this study are preliminary design choices that require experimental validation in future work. Furthermore, compared with the strengths of radar-based approaches, a key limitation of camera-captured images is the difficulty of reliably detecting objects under nighttime lighting or adverse weather conditions. Recent studies have addressed this challenge by collecting data under more diverse environmental conditions and by applying robust augmentation techniques to improve model performance. Additionally, this work represents an early-stage, proof-of-concept study; we focused on a single, commonly used 250 cc scooter model to establish the foundational braking ratio analysis. However, the front braking ratio function should be modified to enhance its generalizability across different motorcycle types, given the wide variations in mass distribution, center-of-gravity profiles, wheelbase geometry, and braking characteristics, all of which may influence the optimal front braking ratio.

The following improvements are required to make the proposed system ready for real-world application: (1) the robustness of the deep-learning-based object detection model should be improved by collecting a larger and more diverse dataset; (2) the front braking ratio should be experimentally measured to determine the optimal braking performance in practice; and (3) a physical actuator should be designed that can control the braking intensity and front braking ratio in real time. In future work, AI-powered detection and prevention of motorcycle collisions with obstacles will be addressed. For more complex scenarios that more closely reflect real-world applications, future research should consider the varying braking intensity, rider override, and brake-release behavior in greater detail, combined with experimental validation.

## 7. Conclusions

This study developed and successfully implemented an automatic braking assistance system incorporating a deep-learning-based object detection model and mechanisms for selecting braking intensity and front braking ratio. The YOLOv12s model exhibited high performance in the detection of car door states, showing precision, recall, and mAP values of 90.5%, 80.6%, and 87.8%, respectively. The braking intensity of the proposed system was determined based on the opening state of the car door, time-to-collision values, and the rider’s braking action, with levels set at 0%, 25%, 50%, or 100% of the maximum braking intensity. The adjustable front braking ratio ensures that the optimal braking performance is achieved regardless of the initial braking speed. At an initial braking speed of 60 km/h, the braking stroke of the proposed system was 35.61% and 13.37% shorter (higher performance) than that of systems using fixed front braking ratios of 20% and 60%, respectively. Additionally, the proposed system can respond within a safe time window under the conditions studied. The total system latency is estimated to be approximately 0.5 s, consisting of 33.33 ms for the image capture, 30.58 ms for the AI device inference, and 463 ms for the hydraulic braking system’s mechanical response before full braking force is generated [34], which is shorter than the TTC threshold value (1.5 s). Therefore, the proposed model demonstrates high feasibility in terms of timely response.

## Figures and Tables

**Table 1:** Dataset for the training model.

	Training Set	Validation Set
Images	1190	295
Labels	1921	477
Ratio	80%	20%

**Table 2:** Computer configuration and model training hyperparameters.

Features	Parameters
**Computer configuration**	
CPU	AMD Ryzen 9 5950X 16-core processor 3.40 GHz
RAM	64.0 GB
GPU	NVIDIA GeForce RTX 3080 10 GB
OS	Window 11
Deep learning models	YOLOv12n, s, m, and l
Python	3.12.7
Pytorch	2.5.1
Cuda	12.1
**Model training hyperparameters**	
Epoch	300
Image size	640 × 640
Batch size	8
Hue/Saturation/Value (HSV)	0.015/0.7/0.4
Translate	0.1
Mosaic	1.0
Flip left-right	0.5

**Table 3:** The performance of the four different-scale YOLOv12 models.

Model	Parameters (M)	Precision	Recall	mAP@0.5	Inference Time (ms)
YOLOv12n	2.6	0.906	0.753	0.839	24.0
YOLOv12s	9.3	0.905	0.806	0.878	24.3
YOLOv12m	20.2	0.859	0.843	0.875	28.9
YOLOv12l	26.4	0.878	0.767	0.845	41.0

**Table 4:** The performance of the YOLOv12s models with and without augmentation.

Model	Precision	Recall	mAP@0.5
YOLOv12s without augmentation	0.818	0.676	0.762
YOLOv12s with augmentation	0.905	0.806	0.878

**Table 5:** Formalized safety mechanism for the automatic braking assistance system.

Time-to-Collision	Opening States of the Car Door	Rider Braking Response	Automatic Braking Assistance System Response
>3 s	Small	Yes/No	Light (green/yellow/red)
	Medium		
	Large		
3 s to 1.5 s	Small	Yes/No	Light (green)
	Medium	Yes	Light (yellow)
		No	Light (yellow) and 25% braking
	Large	Yes	Light (red)
		No	Light (red) and 25% braking
≤1.5 s	Small	Yes or no	Light (green)
	Medium	Yes	Light (yellow)
		No	Light (yellow) and 50% braking
	Large	Yes	Light (red)
		No	Light (red) and 100% braking

**Table 6:** Motorcycle specifications for the simulation problem.

Specifications	Parameters
Motorcycle type	Scooter
Engine	250 cc, 22 hp
Scooter mass	173 kg
Brake system	Hydraulic disk, separate brake
Suspension system	Telescopic fork and unit swing arm

## Data Availability

Data are contained within the article.
